# Idiopathic Spinal Epidural Lipomatosis Causing Cauda Equina Syndrome

**DOI:** 10.5811/cpcem.2017.6.34778

**Published:** 2017-10-03

**Authors:** John B. Bushkar, Lacey P. MenkinSmith, Diann M. Krywko

**Affiliations:** * Medical University of South Carolina, Charleston, South Carolina; † Medical University of South Carolina, Department of Emergency Medicine, Charleston, South Carolina

## Abstract

Spinal epidural lipomatosis (SEL) is a rare condition defined by the hypertrophy of adipose tissue in the spinal epidural space, often resulting in compression of nerves in the region affected.^1^ This case describes a 64-year-old man who presented with cauda equina syndrome. Magnetic resonance imaging of the spine revealed extensive SEL of the lumbar spine. He underwent decompression and fusion with subsequent improvement of symptoms. This is one of the few cases reported of lumbar SEL in a non-obese patient in absence of long-term corticosteroid usage. We review possible etiologies.

## INTRODUCTION

Spinal epidural lipomatosis (SEL) is a rare condition described by the hypertrophy of adipose tissue in the spinal epidural space, often resulting in compression of nerves in the region affected.^1^ This overgrowth of fatty tissue may cause spinal cord compression and in limited cases lead to cauda equina syndrome (CES).^2^ SEL most commonly occurs in the thoracic spine; however, it can also occur in the lumbar region. In 75% of SEL cases, the patient has a history of long-term corticosteroid use, defined by a mean of 30–100 mg per day for a duration of 5–11 years.^3^

In addition to long-term corticosteroid use, etiologies include obesity, Cushing’s disease, Cushing’s syndrome, pituitary prolactinoma, and hypothyroidism.^3^ Benign symmetrical lipomatosis, also known as Madelung’s disease, is a disorder attributed to chronic alcoholism that involves fat deposition throughout the neck, shoulders, and arms. In type 1 disease, these infiltrative, non-encapsulated lipomas will be limited to the nape of the neck and supraclavicular regions.^4^ To date, no cases of SEL have been attributed to Madelung’s disease.

We describe here a unique case of SEL confined to the lumbar region leading to CES in a normal-weight patient with history of recurrent non-Hodgkin lymphoma (NHL), chronic alcoholism, and a neck lipoma.

## CASE REPORT

A 64-year-old male presented with complaints of one month of falls and two years of progressive lower extremity weakness, urinary incontinence, bowel incontinence and back pain without numbness or erectile dysfunction. Past medical history included NHL, hypertension, myocardial infarction, right middle cerebral artery (MCA) stroke with residual left-sided weakness, and chronic alcohol abuse. The patient admitted to drinking a pint of liquor per day, with recent rehab and cessation from drinking for one month prior to presentation.

After his diagnosis of NHL in 1985, the patient had received a drug regimen of rituximab, cyclophosphamide, hydroxydaunomycin, and oncovin, in addition to prednisone (collectively known as RCHOP). This resulted in remission until recurrence of disease in 2005, wherein treatment with chemotherapy and surgical resection was initiated. He received three of the six recommended cycles of RCHOP at that time. Other than those two remote time periods, the patient denied receiving any other corticosteroids.

On presentation, the patient had a blood pressure of 141/89 mmHg, pulse 82 beats per minute, temperature 36.9°C, respiratory rate 18 breaths per minute, and oxygen saturation 97%. He had a body mass index (BMI) of 25 kg/m2. The patient was alert and oriented to person, place, and time and was normocephalic and atraumatic. His cardiovascular exam had a regular rate and rhythm with no murmurs, rubs, or gallops. Pulmonary was clear to auscultation with no wheezes, rhonchi, or rales. The abdomen was soft, non-tender, non-distended. He had normal fluent speech and there were no noted skin abnormalities. Further examination revealed difficulty with gait – particularly with lifting his feet off the ground, bilateral lower extremity weakness that was worse on the left, and mild tenderness to palpation in the lumbar spine. The patient was unable to ambulate in the department despite repeated efforts.

**Image 1:**
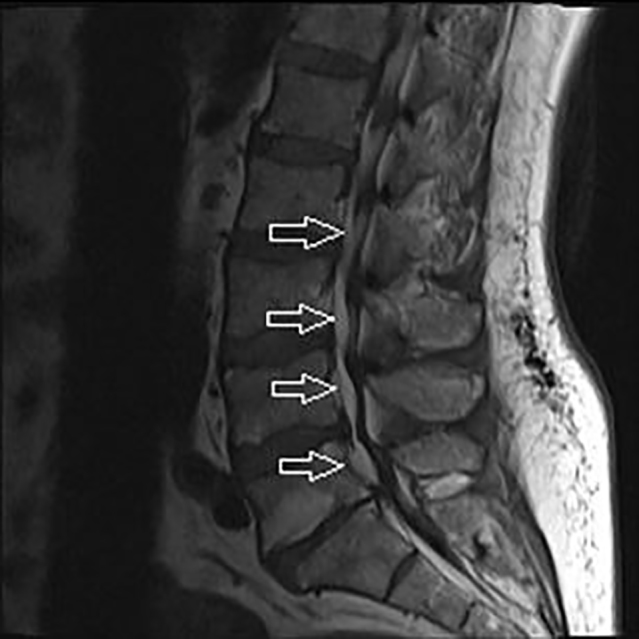
T1 weighted sagittal view of lumbar spine with arrows pointing to epidural lipomatosis.

**Image 2:**
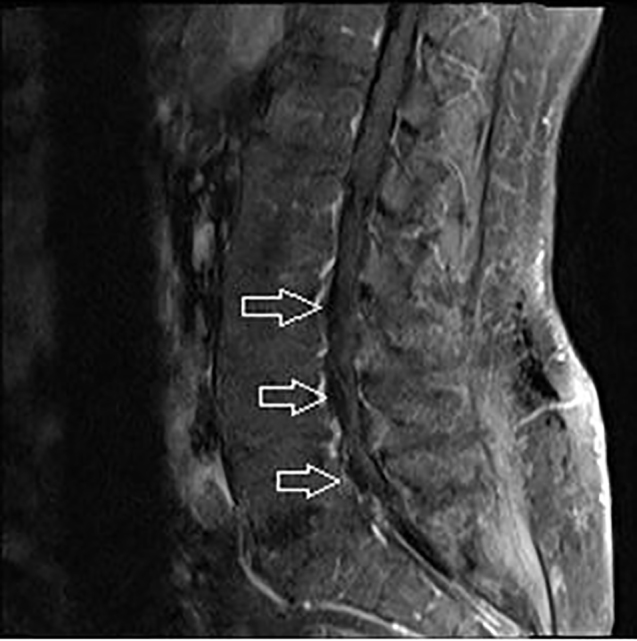
T1 weighted fat suppression sagittal image of lumbar spine with arrows pointing to lipomatosis (Blood would show bright in this view).

**Image 3:**
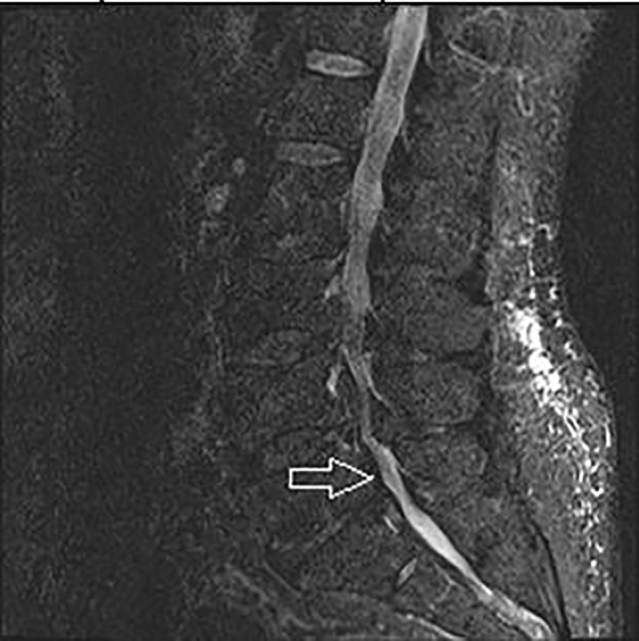
Short T1 inversion recovery image of lumbar spine with arrow pointing to lipomatosis.

**Image 4:**
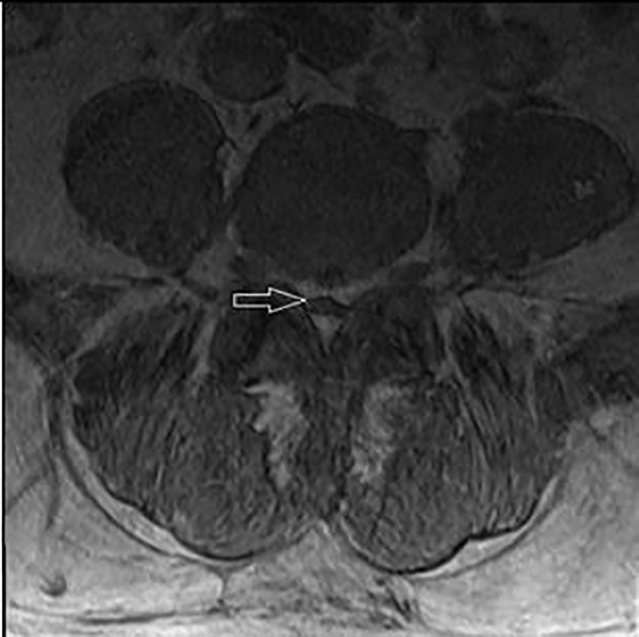
T1 weighted axial view of lumbar spine with arrow pointing to lipomatosis.

Complete blood count, basic metabolic panel, hepatic function, urinalysis, erythrocyte sedimentation rate, and C-reactive protein were all unremarkable. Non-contrast computed tomography (CT) of the head showed the chronic right MCA infarct with no acute processes. Magnetic resonance imaging (MRI) of the cervical spine revealed a 7×3 cm lipomatous lesion in the right side of the neck corresponding to a fat-containing mass noted to be enlarged from a prior CT in 2010 (Images 1–4). MRI of the thoracic spine displayed stable postsurgical and radiation changes with no epidural masses. MRI without and with intravenous contrast of the lumbar spine revealed progressive severe proliferation of epidural fat from Lumbar 3 (L3) to Sacral 1 (S1) segments consistent with SEL.

Neurosurgery was consulted and the patient was taken to the operating room where he underwent decompression and fusion. The neurosurgeon performed laminectomies of L3-S1, noting “extensive lipomatosis throughout.” The adipose tissues were removed and the nerve roots were extensively decompressed. In some areas, the ligamentum flavum and bone were attached to the dura. A cerebral spinal fluid (CSF) leak, discovered upon removal of some areas, was repaired. Rods were inserted and the wound was irrigated and closed using vicryl sutures.

**CPC-EM Capsule**

What do we already know about this clinical entity?

Spinal epidural lipomatosis is a rare cause of cauda equina syndrome in patients with long-term steroid use, obesity, Cushing’s syndrome, prolactinoma, or hypothyroidism.

What makes this presentation of disease reportable?

This is a unique presentation of cauda equina syndrome secondary to spinal epidural lipomatosis in a patient without any currently known risk factors.

What is the major learning point?

Spinal epidural lipomatosis may result in spinal cord compression. Though there are known risk factors, it may also present idiopathically.

How might this improve emergency medicine practice?

This case raises awareness of spinal epidural lipomatosis as an etiology to consider in patients with and without risk factors presenting to the ED with cauda equina syndrome.

Recovery from the surgery went well at first, with the patient making gains in movement and strength. He was noted to be motivated and worked aggressively with nursing, physical therapy (PT), and occupational therapy (OT). The patient did have a brief setback five days into recovery when he became delirious, had difficulty working on his goals, and began refusing rehabilitation with PT. Upon resolution of the delirium, he reengaged with PT/OT. His sitting balance improved and he was able to bear weight on his bilateral lower extremities for transfers. The patient was recommended by therapy for sub-acute rehab and was discharged 15 days post-op.

## DISCUSSION

The first case of SEL was published in 1975 on a patient receiving long-term corticosteroids for renal transplantation.^5^ Since that time, very few cases of this entity have been reported in the literature. More recently, an isolated case was reported in the emergency medicine literature, with the cause attributed to obesity.^6^

Each of the known causes of SEL (corticosteroid use, obesity, Cushing’s disease, Cushing’s syndrome, pituitary prolactinoma, and hypothyroidism^3^) are associated with abnormalities of adipose distribution. For example, long-term corticosteroid use causes weight gain, a moon face, and increased abdominal fat.^3^ Cushing’s disease results in the classic redistribution of fat to the face and neck. Obesity, which is also one of the top etiologies, presents with excess fat distribution throughout the body. Another condition known as Madelung’s disease (benign symmetrical lipomatosis) is associated with chronic alcoholism. It causes redistribution of fat to the neck and shoulders as well; however, it is not a known etiology of SEL.^4^

The patient in this case had none of the known causes of SEL, as he had no recent or long-term corticosteroid use, endocrine disorder, or obesity. The threshold for determining obesity-related SEL is a BMI greater than 28 kg/m.^2^. From 28–35 kg/m^2^ there is a linear increasing relationship between BMI and SEL risk.^7^ The patient in this case had a BMI of 25 kg/m^2^ and would not meet obesity criteria for the disease. Further, his SEL presented in the lumbar spine. The rarely encountered lumbar SEL is more likely to be obesity-related or idiopathic. In contrast, the majority of SEL cases involve the thoracic spine and are related to long-term corticosteroid treatment or endocrinopathy.^8^

The patient in this case had a history of chronic alcohol abuse. Chronic alcohol consumption has a well-documented link to fat redistribution. The most notable adipose pathology associated with alcohol consumption is alcoholic fatty liver disease. Excess alcohol consumption leads to increased adipose tissue lipolysis and ectopic fat deposition into the liver and other peripheral organs of the body. Adipokines, such as leptin and adiponectin, play a vital role in the regulation of metabolism within various tissues like the brain, skeletal muscle, and liver. These proteins have been shown to be implicated in patients with alcoholic lipodystrophy and hepatic steatosis.^9^ Kim S-S et al. present a case discussion of a patient with chronic alcoholism and SEL, suggesting a possible link between chronic alcohol abuse, fat redistribution, Madelung’s disease, and the development of SEL.^10^ However, that particular patient had no appreciable signs of Madelung’s disease.

## CONCLUSION

In the absence of corticosteroid use, obesity, and other endocrine diseases, our patient remains without a definitive cause for his rare lumbar SEL. While he did have remote, short-term exposure to steroids with his reoccurrence of NHL in 2005, he was not exposed for the typical duration that is commonly associated with SEL.^3^ The patient also had a BMI of 25 kg/m^2^, placing him in the non-obese weight category. Lastly, this patient did have a large lipoma of the neck in the context of chronic alcohol abuse, making Madelung’s disease a likely possibility. However, no biopsy or resection of the lesion was performed to verify the diagnosis. Although they are not known causes of SEL, Madelung’s disease and chronic alcoholism can cause redistribution of fat throughout the body similar to the other known etiologies of SEL. As such, there is a possibility of Madelung’s disease and chronic alcohol abuse being the causative etiology for the fat distribution causing this patient’s lumbar type of SEL.
